# Widespread myeloid sarcomas in a patient in morphological remission in the bone marrow

**DOI:** 10.1002/jha2.145

**Published:** 2021-02-16

**Authors:** Jennifer Vidler, Kate Fletcher, Sukanya Gogoi, Paraskevi Gkreka, Jonathan R. Salisbury, M Mansour Ceesay

**Affiliations:** ^1^ Department of Haematology King's College Hospital NHS Foundation Trust Princess Royal University Hospital Orpington UK; ^2^ Department of Histopathology King's College Hospital NHS Foundation Trust London UK

A 60‐year‐old woman previously diagnosed with acute myeloid leukaemia (AML) with fibrosis (normal karyotype, *NPM1*‐mutated, *FLT3*‐ITD negative, *TET2* mutations (two variants) and *SRSF2* mutations detected on our myeloid gene panel) presented with worsening pain and swelling in her right arm and leg. She was unable to stand due the leg pain.

She was refractory to standard daunorubicin and cytarabine induction chemotherapy but achieved complete morphological and immunophenotypic remission with FLAG Ida (fludarabine, cytarabine, granulocyte colony stimulating factor, and idarubicin) (Figure 1). Magnetic resonance imaging showed a mass within the humeral bone and overlying muscle. Widespread metabolically active bony lesions (blue arrows) were found on ^18^F‐fluorodeoxyglucose positron emission tomography and an associated pathological fracture of the right hip (red arrowhead) (Figure 2). Biopsy of the humeral lesion revealed myeloid sarcoma, as did histology of the femoral head (Figure 3). She underwent orthopaedic surgery and received radiotherapy (400 cGy in two fractions) to the arm and leg lesions followed by venetoclax plus a continuous infusion of low‐dose cytarabine. Unfortunately, she failed to respond to this combination and eventually died from her disease.

**FIGURE 1:**
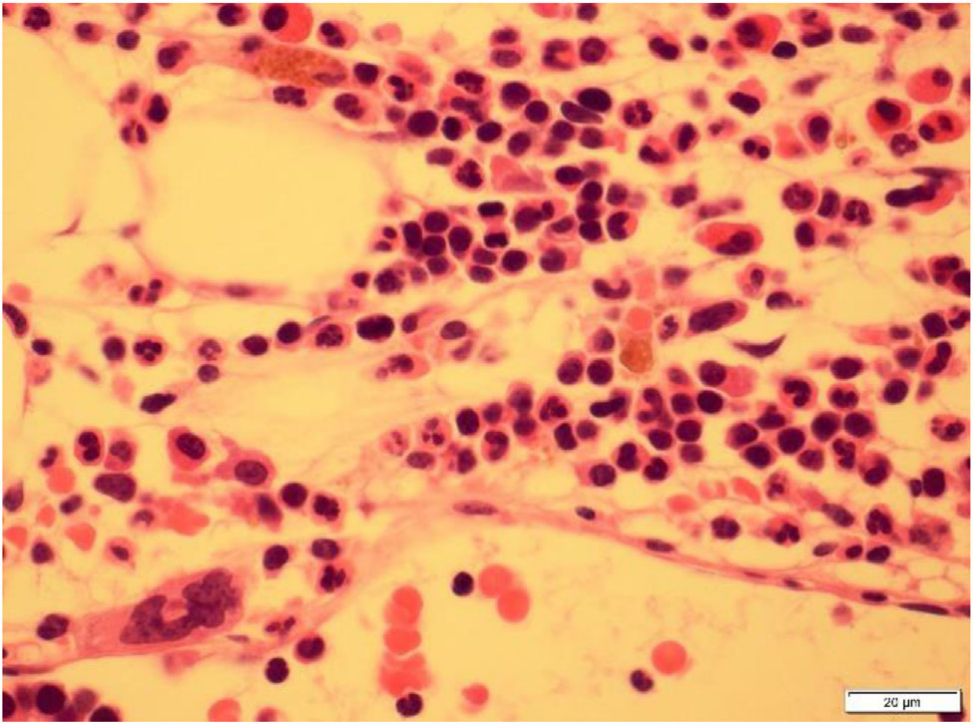
Bone marrow trephine biopsy showing a fibrotic morphological remission from AML

**FIGURE 2:**
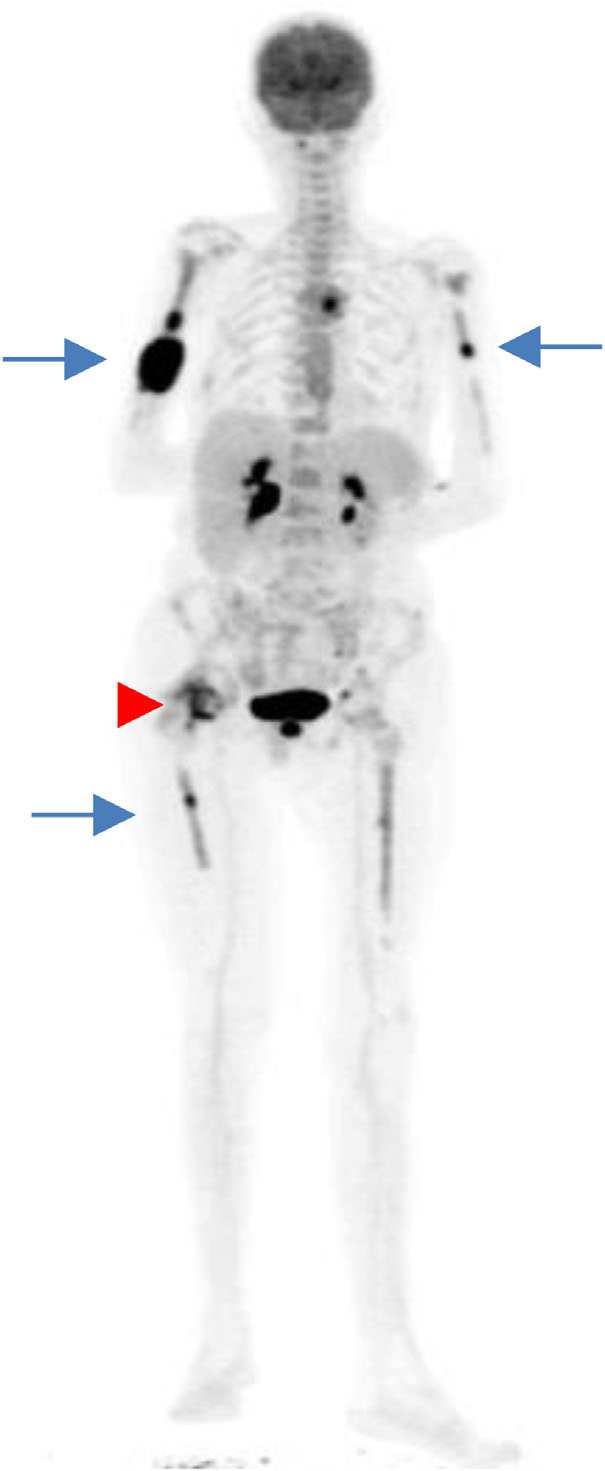
^18^FDG PET‐CT scan showing multifocal myeloid sarcomas

**FIGURE 3:**
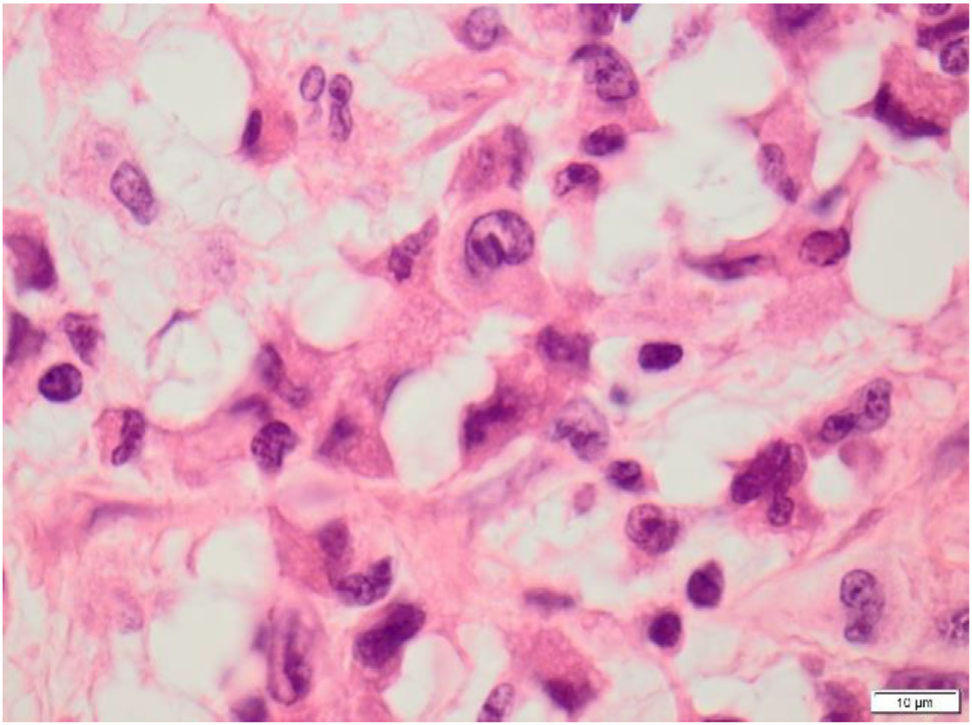
Histology showing myeloid sarcoma of the femoral head

Myeloid sarcomas are extramedullary variants of myeloid malignancies. This patient had widespread extramedullary disease, whilst the bone marrow was in morphological remission. This highlights the need for clinicians to be wary of unusual presentations of relapsed disease in patients with AML.

## AUTHOR CONTRIBUTIONS

JV and MC provided the imaging and wrote the manuscript; PG, KF and SG provided the clinical data; and JS provided the histology images.

